# Factors Associated With Virtual Reality Sickness in Head-Mounted Displays: A Systematic Review and Meta-Analysis

**DOI:** 10.3389/fnhum.2020.00096

**Published:** 2020-03-31

**Authors:** Dimitrios Saredakis, Ancret Szpak, Brandon Birckhead, Hannah A. D. Keage, Albert Rizzo, Tobias Loetscher

**Affiliations:** ^1^Cognitive Ageing and Impairment Neurosciences Laboratory, School of Psychology, Social Work and Social Policy, University of South Australia, Adelaide, SA, Australia; ^2^Division of Health Services Research, Department of Medicine, Cedars-Sinai Health System, Los Angeles, CA, United States; ^3^Institute for Creative Technologies, University of Southern California, Los Angeles, CA, United States

**Keywords:** cybersickness, simulator sickness, head-mounted display, virtual reality, virtual environment

## Abstract

The use of head-mounted displays (HMD) for virtual reality (VR) application-based purposes including therapy, rehabilitation, and training is increasing. Despite advancements in VR technologies, many users still experience sickness symptoms. VR sickness may be influenced by technological differences within HMDs such as resolution and refresh rate, however, VR content also plays a significant role. The primary objective of this systematic review and meta-analysis was to examine the literature on HMDs that report Simulator Sickness Questionnaire (SSQ) scores to determine the impact of content. User factors associated with VR sickness were also examined. A systematic search was conducted according to PRISMA guidelines. Fifty-five articles met inclusion criteria, representing 3,016 participants (mean age range 19.5–80; 41% female). Findings show gaming content recorded the highest total SSQ mean 34.26 (95%CI 29.57–38.95). VR sickness profiles were also influenced by visual stimulation, locomotion and exposure times. Older samples (mean age ≥35 years) scored significantly lower total SSQ means than younger samples, however, these findings are based on a small evidence base as a limited number of studies included older users. No sex differences were found. Across all types of content, the pooled total SSQ mean was relatively high 28.00 (95%CI 24.66–31.35) compared with recommended SSQ cut-off scores. These findings are of relevance for informing future research and the application of VR in different contexts.

## Introduction

Despite advancements in virtual reality (VR) technology, many people still report experiencing simulator sickness symptoms from its use (Rebenitsch and Owen, 2016; Gavgani et al., 2017; Duzmanska et al., 2018; Guna et al., 2019). Characterizing and quantifying these symptoms is challenging, as several factors are at play including a diverse range of technologies; the use of inconsistent terminology for sickness from using virtual environments; little consensus on the biological mechanisms of symptoms; the diverse range of VR content; along with user characteristics such as age and sex (Hale and Stanney, 2014). Identifying factors that increase the occurrence of simulator sickness becomes necessary with the increased use of VR for rehabilitation, industry training and gaming/entertainment consumers (Gallagher and Ferrè, 2018; Powell et al., 2018; Wang et al., 2018).

Side effects from virtual environment usage has been referred to by many terms including simulator sickness (Kennedy et al., 1993), cybersickness (LaViola, 2000) and VR sickness (Kim et al., 2018). The term simulator sickness originated from the early use of flight simulators in the military (Kennedy et al., 1993), and is still currently used in research using modern HMD technology (Tyrrell et al., 2018; Ziegler et al., 2018). Cybersickness, originally used to describe side effects from use of virtual environments (McCauley and Sharkey, 1992), has often been mentioned in studies using a variety of technologies including flat screen displays and head-mounted displays (HMD) (Rebenitsch and Owen, 2016). The term VR sickness has typically been used in studies using HMDs (Cobb et al., 1999; Kim et al., 2018). Thus, diverse terminology is often used interchangeably across the virtual environments literature.

This current review focuses on adverse symptoms from HMD use, hence the term “VR Sickness” will be referred to as the symptoms (and their severity) typically reported in the literature from HMD use. The term “motion sickness” will be used to refer to more general reporting of symptoms from motion environments (e.g., air, land, or sea travel), not specific to HMDs, where symptoms can differ. For example, nausea can be more severe in seasickness, compared with simulator use (Kennedy et al., 2010). Symptomatology of sickness also differs between technologies. Compared with simulators, HMDs have been reported to produce higher symptoms related to nausea, dizziness and blurred vision (Kennedy et al., 2003).

Measures of VR sickness are a fundamental part of establishing prevalence and symptomatology in virtual environments. The Simulator Sickness Questionnaire (SSQ) (Kennedy et al., 1993), originally developed for measuring motion sickness in simulators, is the most commonly used measure of sickness in virtual environments (Rebenitsch and Owen, 2016). Alternate measures, such as the *Virtual Reality Symptom Questionnaire*, which was specifically developed for HMDs (Ames et al., 2005) or the *Virtual Reality Sickness Questionnaire* (Kim et al., 2018) have yet to be widely adopted. Single item assessments that are easy to administer and monitor symptoms during VR exposure (Bos et al., 2005; Keshavarz and Hecht, 2011) are commonly used, but do not provide comprehensive measurements of the symptoms of VR sickness. Very few studies report on the use of objective physiological measures (e.g., heart rate, skin conductance, electroencephalograms, eye blink rate, and electrogastrogram) that do not rely on individual self-report data (Kim et al., 2005; Dennison et al., 2016).

Recent advances in HMD technology (field of view, resolution, framerate, and ergonomic factors) have increased the levels of immersion and realism that may have an influence on the occurrence of VR sickness (Nichols, 1999; Lee et al., 2017; Kourtesis et al., 2019). For example, if an image is clear and tracking of movement is accurate, there may be fewer sensory conflicts, and that could lead to a reduction in VR sickness symptoms (White et al., 2015; Shin et al., 2016; Ray et al., 2018). However, an increase in the field of view may also increase risk of VR sickness (Fernandes and Feiner, 2016). Despite the improvements in HMD technology, a recent review suggests that the prevalence of VR sickness is still problematic (Rebenitsch and Owen, 2016). In addition to this, Kourtesis et al. (2019), in their review found that although recent hardware features have been an important factor in reducing VR sickness, software features also need to be taken into consideration.

The VR content delivered to users can induce or even reduce VR sickness. A rollercoaster ride may be more likely to induce VR sickness to the level of severity where users will request to discontinue the experience. For example, almost 67% of participants in a study using a rollercoaster virtual environment were unable to complete an exposure time of 14 min (Nesbitt et al., 2017). In contrast, content consisting of low amounts of motion may be less likely to induce VR sickness (Guna et al., 2019), as well as in cases where head movement in a fixed position is concordant with what the user would experience in the real world (Rizzo and Koenig, 2017).

Length of time exposed to a virtual environment may also influence likelihood and severity of VR sickness (Duzmanska et al., 2018). Significant correlations have been found between exposure time and VR sickness, with longer exposure times increasing risk of VR sickness (Stanney et al., 2003). For example, research measuring VR sickness at multiple time points found symptoms increased at 2-min increments, with the highest VR sickness scores measured in the final trial at 10 min (Moss and Muth, 2011). In contrast, a recent review has found that some people may build up a resistance or adapt over time to VR sickness, particularly over multiple sessions (Duzmanska et al., 2018). Although content and duration are significant contributing factors that may increase the likelihood of sickness symptoms, the user also needs to be taken into consideration.

User characteristics adds another layer of complexity in understanding the relationship between hardware, content and VR sickness. Research on sex and age, have generated mixed findings when it comes to the likelihood of sickness from VR (Cheung and Hofer, 2002; Benoit et al., 2015; Munafo et al., 2017; Arcioni et al., 2018). In reference to age, physiological differences over the lifespan (i.e., visual, vestibular senses) (Bermúdez Rey et al., 2016) may influence the occurrence of VR sickness and symptom profiles. For example, hormonal differences in females have been reported to influence and likely to be a factor in increased rates of VR sickness (Clemes and Howarth, 2005). Moreover, females can have a smaller interpupillary distance (Fulvio et al., 2019) and some HMDs may not be able to be adjusted accordingly therefore creating eye strain and general discomfort. Thus, it is important to increase the understanding of the relationship between these user characteristics and VR sickness.

Previous reviews (Rebenitsch and Owen, 2016; Duzmanska et al., 2018; Kourtesis et al., 2019) have focused on temporal or technological aspects of VR sickness. To date, none of the reviews on VR sickness have systematically evaluated VR content and user characteristics in a meta-analysis. The primary aim of this systematic review is to examine if VR sickness symptoms measured with the SSQ using HMDs are influenced by different factors. More specifically, factors that will be examined in this review are content, the amount of visual stimulation (motion of virtual environment), whether a person is stationary or moving in the virtual environment and time. As the SSQ consists of three grouped factors (nausea, oculomotor, and disorientation), a summary of the most common symptoms using HMDs will be provided. Studies with the intention of inducing or not inducing VR sickness will also be compared. A secondary aim is to examine the influence of user characteristics (i.e., age and sex) on SSQ scores and dropout rates.

## Methods

### Search Strategy

In accordance with the Preferred Reporting Items for Systematic Reviews and Meta-Analysis (PRISMA) statement (Liberati et al., 2009), a systematic literature search was conducted to reveal journal and conference papers related to VR sickness from using HMDs. This review included the following search terms: virtual reality OR virtual environment^*^ OR VR OR VR headset OR virtual reality headset OR head-mounted display OR HMD OR helmet mounted display AND cybersickness OR motion sickness OR simulator sickness OR visually induced motion sickness OR virtual reality induced motion sickness OR virtual reality induced symptoms and effects OR virtual reality sickness OR visual-vestibular OR nausea OR aftereffect^*^ OR after effect^*^ OR VIMS. No limiters were inserted in the database searches.

This search was carried out on the 10th October 2018 in the six databases: Cochrane Library, IEEExplore, Medline, PsycINFO, Scopus, and Web of Science. Terms were mapped to subject headings. Both journal and conference articles were *included* in this review if: participants used a head-mounted display (HMD); VR sickness was measured using the SSQ; articles were peer-reviewed and complete (i.e., includes a full paper, not just an abstract or poster presentation); the text was in English or had been translated for publication. Papers were *excluded* if they: used augmented reality (AR) or see-through displays; were reviews, dissertations, abstracts or poster presentations; used prototype HMD devices; and were case studies. Papers that included clinical samples were also excluded, however, if the study included a healthy control group, this data was included. Eligibility of studies was assessed by two independent reviewers (DS and AS).

Papers were included if they supplied mean data for the SSQ (either subscales or total scores), if no mean data was supplied they were still included in the dropout analysis if they indicated drop out rates. If papers supplied mean scores without standard deviations, authors were contacted to supply the standard deviations. Current contact details were searched for online in each case. A follow-up email was sent to authors that did not respond to the initial email. If the authors did not respond to the second email the paper was excluded. The calculation of subscale and total SSQ scores required weighting. Subscales are weighted as follows; nausea 9.54; oculomotor 7.58; and disorientation 13.92, while total scores can be calculated by multiplying unweighted subscale scores by 3.74 (Kennedy et al., 1993). This can create some confusion at times, and there were instances where researchers calculated the scores differently. For example, multiplying the weighted subscale scores by 3.74 thereby producing inflated total scores. There were also instances where the total SSQ scores did not match the subscale scores, the same contact procedure was followed for these papers as per the missing standard deviations.

**Figure 1:**
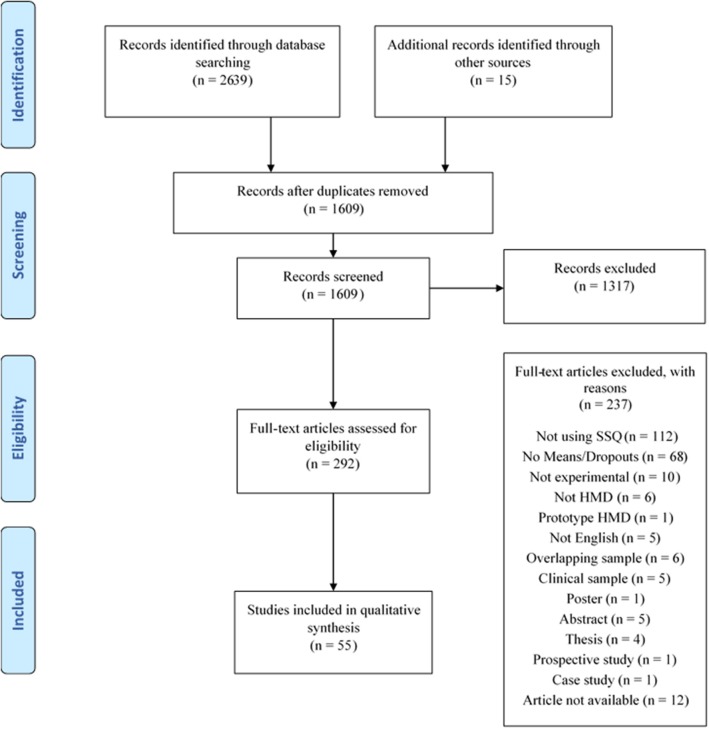
The article selection and screening process using the PRISMA flow diagram (Liberati et al., 2009).

Figure 1 shows the results of the electronic search and article selection as per PRISMA guidelines (Liberati et al., 2009).

### Statistical Approach

Comprehensive Meta-Analysis (CMA) Version 3 (Borenstein et al., 2013) was used to conduct meta-analyses. A random effects model was used to calculate pooled effect estimates with 95% confidence intervals. In studies reporting multiple experiments within groups, these means were merged in CMA to produce one mean per study. In studies reporting multiple experiments between groups, these means were calculated separately for each experiment. Pooled means were calculated for all factors separately on each subscale of the SSQ. Pooled means were also calculated for all factors separately for the total SSQ score. Differences between sub-factors within each factor were assessed using the *Q*-test based on analysis of variance (Borenstein et al., 2011). The *Q*-value for the between group analyses corresponded to the weighted sum of squared deviations of the subgroup means about the grand mean. *P*-values were obtained by comparing the *Q*-values with a chi-squared distribution with degrees of freedom equal to the number of subgroups minus one (Borenstein et al., 2013). A *p*-value lower than 0.05 was assumed to indicate a significant statistical difference of SSQ scores between the sub-factors. A correlation was performed between the percentage of females in studies and total SSQ scores as breakdowns for sex of means for the SSQ scores were not supplied in most studies.

### Operationalisation of Factors Being Examined

All factors were operationalised and independently reviewed by DS and AS. Any disagreements were resolved by discussion.

### Content

**Figure 2:**
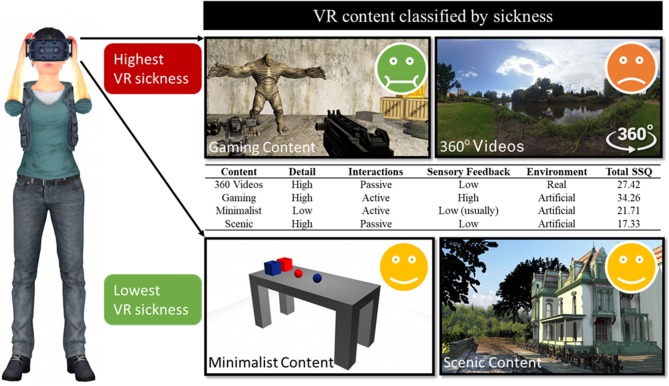
Content characteristics and participant's sickness response. Emoticons indicate participant level of discomfort according to total Simulator Sickness Questionnaire (SSQ) scores averaged across all studies.

Four types of content were categorized in studies included for analysis; 360 videos; gaming content; minimalist content; and scenic content. User interaction and environmental features differed for each category. The 360 videos included content captured with a 360 camera or video taken that allowed a 360 view of the virtual environment. Gaming included high detailed content where the user could actively interact and perform tasks in the virtual environment including off-the-shelf games and content developed by researchers. Minimalist content consisted of basic shapes or minimal textures, with typically simple interactions. Scenic content included detailed environments, for example, a landscape or cityscape with no or simple interaction by the user. See Figure 2 for a summary of content characteristics.

### Visual Stimulation

All studies were categorized based on the amount of visual movement within the content regardless of user-directed movement, such as locomotion and head movement. Low visual stimulation included content with slow visual changes, while high visual stimulation included content with fast visual changes.

### Locomotion

Locomotion refers to how a user navigates in the virtual environment. For the analysis in this review, locomotion was classified as either stationary, controller-based movement, or physically walking. With stationary content, the user does not move in the virtual environment. Two moving categories were included; controller and walking. Controller-based movement included the following navigation methods; flying; controller-based walking; teleporting and driving, therefore any movement for navigation by the user. Walking included the following physical movements; walking; walking in place and walking on a treadmill. The two categories of moving were used as physically walking has been found to reduce the incidence of VR sickness compared to controller-based navigation (Chance et al., 1998).

### Time

Sickness in virtual environments has been found to increase after 10 min in HMDs and simulator studies (Min et al., 2004; Moss and Muth, 2011). Thus, time was categorized into three intervals of 10 min: <10 min, ≥10 min, or ≥20 min.

### VR Sickness Condition

Studies that explicitly set out to increase/decrease the occurrence of VR sickness or measured VR sickness as a secondary aim, were categorized into two conditions: induce, and not induce.

### User Characteristics

The user characteristic of age was categorized into a mean age of <35 years old and ≥35 years old. This cut-off was used to correspond with theories of both sensory conflict and postural instability. For example, vestibular function involved in the sensory conflict theory starts to decline around the age of 40 (Bermúdez Rey et al., 2016). With relevance to the postural instability theory, changes in altered postural balance have been reported to commence at the ages of 30–39 (Era et al., 2006).

Mean breakdowns by sex were not supplied in most SSQ studies. Therefore, a correlational analysis was performed looking at the proportion of sex (females) in studies with total SSQ mean scores. This approach aimed to give an approximation due to the lack of available data, a positive correlation in this analysis will indicate higher susceptibility of VR sickness in females.

### Dropouts

Dropouts in this review refer to participants that exited an experiment due to VR sickness.

## Results

A total of 2,654 publications were identified through the search. A snowballing strategy was used to identify an additional 15 articles for inclusion. These publications were imported into EndNote where 1,045 duplicates were removed. The remaining 1,609 articles were sent to Covidence systematic review management software (Covidence, 2019) for title and abstract screening, which identified 292 articles for full-text screening. A further 237 articles were excluded as outlined in Figure 1. Authors were contacted for 15 papers as per the procedure described in the methods section if mean scores were supplied without standard deviations (10), or if scores did not appear to be weighted correctly (5). A total of 54% of authors replied with 20% supplying raw data to enable calculation of SSQ scores. Hence, 55 publications were identified through the systematic review process and listed in Table 1.

**Table 1:** Summary of included articles.

**Authors**	***n***	**Age *mean (SD)***	**Content**	**Visual stimulation**	**Locomotion**	**Time (minutes)**	**Dropout%**	**Condition/ Group**	**Nausea**	***(SD)***	**Oculomotor**	***(SD)***	**Disorientation**	***(SD)***	**Total**	***(SD)***
Arcioni et al. (2018)	21 (14F, 6M)	28.4 (10.1)	Minimalist	High	Stationary	<10	n/a n/a	Compensated Inverse	19.00 20.00	(20.00) (18.00)	19.00 22.00	(14.00) (19.00)	28.00 31.00	(37.00) (30.00)		
Bessa et al. (2016)	63 (32F, 31M)	21.49 (3.85)	360	High	Controller	<10	n/a n/a	2D 3D	2.73 2.94	(4.42) (4.50)	10.83 9.62	(12.59) (9.24)	17.90 12.31	(19.76) (17.31)	11.22 9.21	(11.41) (9.82)
Brooks et al. (2017)	26 (3F, 19M)	31.59 (7.72)	360	High	Controller	≥20	4								22.91	(2.31)
Budhiraja et al. (2017)	15 (3F, 12M)	18−26 (range)	Game	High	Controller	≥10	12	Rotation blurring							42.14	(27.61)
	15 (3F, 12M)						n/a	No rotation blurring							51.36	(34.67)
Carnegie and Rhee (2015)	20 (6F, 14M)	18−50 (range)	Scenic	Low	Controller	≥20	30 n/a	DoF Disabled DoF Enabled	8.00 4.98	(6.19) (5.78)	5.64 3.74	(5.02) (4.19)	8.83 4.60	(6.64) (4.28)		
Christou and Aristidou (2017)	18 (7F, 11M)	24.00	Game	Low	Controller	<10	17 n/a n/a	Pointing Gaze-directed Teleport	36.00 21.00 10.80	(37.20) (21.10) (14.40)	23.80 18.70 13.60	(25.90) (20.60) (17.50)	39.00 29.70 24.10	(46.80) (33.20) (31.30)	36.40 25.40 17.50	(37.80) (25.40) (19.70)
Deb et al. (2017)	21 (11F, 10M)	27.84	Game	Low	Walking	≥20	15		8.63	(11.65)	15.16	(16.95)	11.93	(19.33)	14.07	(16.03)
Dennison and D'Zmura (2017)	15 (4F, 11M)	n/a	Minimalist	High	Stationary	<10	7 n/a	Seated Standing	15.90 34.34	(17.70) (39.79)	19.20 17.18	(18.31) (19.53)	24.13 22.27	(27.01) (37.13)	22.19 27.93	(20.46) (33.27)
Dennison and D'Zmura (2018)	20 (5F, 15M)	18−60 (range)	Game	Low	Controller	≥10	10 n/a	Push No Push	72.61 48.65	(43.92) (47.90)	50.95 29.56	(37.84) (29.35)	77.33 34.80	(73.07) (57.30)	74.38 43.01	(53.23) (46.75)
Dennison et al. (2016)	20 (6F, 14M)	n/a	Game	Low	Controller	≥10	55									
Dorado and Figueroa (2014)	44 (8F, 36M)	22	Game	Low	Controller	<10	n/a	Constant Speed Ramp							17.03	(15.87)
							n/a	Constant Speed Stairs							28.42	(22.25)
Farmani and Teather (2018)	14 (5F, 9M) 14 (6F,8M)	26.4 26.4	Game	High	Controller	≥20	7 14	Viewpoint Snapping No Viewpoint Snapping								
Frommel et al. (2017)	24 (7F, 17M)	27.04 (4.02)	Game	Low	Controller	≥20	n/a n/a n/a n/a	Fixpoint Free Guided Touchpad	19.08 15.90 27.03 28.62	(27.27) (19.22) (22.27) (27.85)	18.00 16.11 28.43 29.06	(24.77) (23.26) (26.38) (26.98)	26.10 29.00 41.76 47.56	(36.79) (35.53) (36.48) (49.07)	23.38 21.97 35.84 38.34	(29.95) (26.36) (29.22) (34.48)
Fujikake et al. (2009)	10 10	23.6 (2.2) 23.6 (2.2)	Minimalist	High	Stationary	<10	n/a n/a	Conventional 3D New 3D	11.40 10.50	(3.70) (4.40)	18.20 17.40	(4.10) (4.90)	23.70 19.50	(8.80) (6.60)	19.80 18.00	(5.30) (4.90)
Guna et al. (2019)	26 (3F, 23M)	24.75 (5.69)	360	High	Stationary	<10	n/a	Oculus Rift DK1 Action	22.38	(22.08)	26.82	(19.91)	44.44	(41.30)	33.95	(25.28)
							n/a	Oculus Rift DK2 Action	29.72	(29.97)	30.32	(27.79)	54.61	(50.72)	41.28	(35.91)
							n/a	Oculus Rift CV1 Action	29.72	(33.63)	24.49	(24.94)	44.97	(54.46)	35.82	(38.47)
							n/a	Samsung Gear VR Action	26.79	(28.30)	31.20	(25.76)	44.44	(46.92)	37.83	(33.39)
Hutton et al. (2018)	20 (6F, 13M)	26	Minimalist	Low	Stationary		25									
Iskenderova et al. (2017)	31 (6F, 25M)	25.4 (3.3)	Game	Low	Controller	≥10	19 n/a n/a n/a	Placebo (1st trial) Placebo (2nd trial) Alcohol (1st trial) Alcohol (2nd trial)	86.65 81.88 82.92 40.36	(25.55) (42.94) (40.73) (35.93)	37.26 46.11 54.22 35.56	(20.3) (26.54) (33.87) (28.08)	77.72 96.28 97.44 64.24	(42.16) (67.29) (68.66) (61.34)	73.24 80.72 85.44 50.63	(27.59) (45.23) (44.62) (42.33)
Kang et al. (2008)	20 (10F, 10M)	48.7 (10.7)	Game	Low	Controller	<10	n/a		24.50	(31.20)	37.90	(26.10)	76.60	(73.60)	35.80	(31.40)
Karl et al. (2013)	44 (15F, 29M)	29 (10)	Game	High	Controller	≥10	4									
Kesztyues et al. (2000)	22 (12F, 10M)	22−50 (range)	Scenic	Low	Controller		45		12.40	(14.20)	11.70	(12.10)	3.60	(5.40)	14.20	(15.40)
Kim et al. (2017)	11 (6F, 5M) 11 (8F, 3M)	28 (7) 66 (3)	Scenic Scenic	Low Low	Walking Walking	≥20 ≥20	0 0	Healthy young Healthy old							8.30 6.50	(10.50) (13.00)
Kinsella et al. (2016)	120	n/a	360	Low	Stationary	≥10	21									
Kruse et al. (2018)	20 (7F, 13M)	25.75	Minimalist	Low	Walking	≥20	n/a								31.23	(31.30)
Kuiper et al. (2018)	18 (8F, 10M)	25.2 (3.6)	Minimalist	High	Controller	≥10	0		10.60	(12.60)	9.68	(8.14)	21.70	(18.60)		
Lee et al. (2017)	20 (10F, 10M)	25.5 (M) 23.4 (F)	Scenic	Low	Controller Controller Walking	<10	n/a	Gamepad Hand interface Walking Simulator	13.36 12.40 12.89	(14.90) (13.86) (13.92)	26.90 25.39 24.64	(24.15) (19.95) (17.76)	50.11 50.81 43.15	(51.22) (47.87) (37.33)	31.98 31.04 28.80	(28.67) (25.23) (22.03)
Ling et al. (2012)	88 (35F, 53M)	28 (6.3)	Game	Low	Stationary	≥10	n/a								2.40	(13.04)
Llorach et al. (2014)	55 (24F, 31M) 61 (23F, 38M)		Scenic Scenic	Low Low	Controller Walking	≥10 ≥10	13 0	Game controller Position Estimation	38.85 15.37	(36.49) (17.16)	15.16 9.97	(15.16) (10.60)	38.48 18.56	(36.00) (22.25)	32.27 15.93	(29.26) (14.81)
McGill et al. (2017)	18 (18M)	25.1 (4.7)	Game	High	Stationary	≥10	n/a n/a n/a n/a	VR Motion VR VR with Compensation VR with Peripheral	39.20 53.50 58.80 60.40	(29.80) (52.40) (49.90) (49.70)	35.00 37.90 43.00 43.40	(28.00) (33.70) (37.30) (35.30)	57.20 62.60 71.90 72.70	(63.40) (71.50) (72.10) (71.40)	47.90 56.50 63.60 64.60	(41.10) (54.70) (55.90) (53.20)
Merhi et al. (2007)	24 (11F, 13M) 9 (2F, 7M)	22 20	Game	High	Controller	≥20	100 59 89	Experiment 1: Standing Experiment 1: Sitting Experiment 2: Sitting							63.60 58.10 79.40	(49.80) (45.80) (24.70)
Mittelstaedt et al. (2018)	60 (40F, 20M)	25.62 (9.34)	Game	High	Controller		7									
Moss et al. (2008)	10 (8F, 2M)	20.6	360	Low	Stationary	≥10	n/a								31.48	(27.37)
Moss et al. (2011)	22 (11F, 11M)	22.6	360	Low	Stationary	≥20	9									
Moss and Muth (2011)	80 (50F, 30M)	19.5 (18−24 range)	360	Low	Stationary	≥10	9								32.33	(4.35)
Munafo et al. (2017)	36 (18F, 18M) 36 (18F, 18M)	20.7 (.85) 22.7 (3.56)	Game	High	Stationary	≥10	17 44	Experiment 1 Experiment 2								
Neth et al. (2011)	32 (17F, 15M)	27.26	Minimalist	Low	Walking		9									
Papachristos et al. (2017)	30 (26F, 4M)	24.83 (8.9)	Game	High	Controller	<10	n/a	Mobile Oculus	17.17 14.63	(25.80) (11.89)	24.76 32.85	(22.82) (23.39)	51.97 47.33	(65.60) (43.96)	32.91 34.66	(37.43) (25.66)
Parijat and Lockhart (2011)	16 (8F, 8M)	74.18 (5.82)	Scenic	Low	Walking	≥20	n/a								5.93	(2.46)
Pettijohn et al. (2018)	17 (4F, 13M)	37.4 (3.5)	Scenic	High	Stationary	≥20	18 n/a n/a n/a n/a n/a n/a n/a	Profile 1 Active Profile 1 Passive Profile 2 Active Profile 2 Passive Profile 3 Active Profile 3 Passive Posttest Active Posttest Passive	14.31 11.58 20.44 19.76 25.89 23.85 12.95 8.18	(4.33) (2.86) (4.99) (4.63) (5.14) (5.08) (3.41) (3.30)	8.12 10.29 13.54 19.49 20.03 22.20 9.75 10.83	(1.68) (2.59) (3.39) (5.37) (5.43) (5.42) (2.69) (3.05)	4.97 7.95 5.97 9.94 8.95 9.94 3.98 3.98	(2.77) (4.05) (4.05) (4.71) (3.31) (5.54) (1.74) (2.27)	10.95 11.75 16.30 20.04 22.44 22.97 10.95 9.62	(2.46) (2.63) (3.56) (5.14) (4.83) (5.14) (2.40) (2.62)
Pot-Kolder et al. (2018)	95 (47F, 48M)	25.4 (4.6)	Game	Low	Controller	≥20	n/a	Control (Excluded Clinical)	40.60	(37.40)	29.30	(29.30)	54.20	(52.10)	44.90	(39.30)
Pouke et al. (2018)	13 13	n/a	Scenic	Low	Controller	≥10	8 0	High realism No detail	29.35 16.88	(37.25) (11.78)	25.66 15.74	(25.62) (12.17)	40.69 28.91	(49.37) (25.07)	35.10 22.15	(39.21) (15.30)
Ragan et al. (2017)	40 (10F, 30M)	18−34 (range)	Game	Low	Controller	≥10	n/a								51.43	(40.21)
Rupp et al. (2019)	136 (66F, 70M)	19.82 (2.44)	360	Low	Controller	<10	n/a n/a n/a	Cardboard Oculus DK2 Oculus CV1	11.5 6.17 6.17	(0.24) (0.33) (0.31)	14.05 10.26 12.48	(0.38) (0.34) (0.37)	16.38 9.42 7.37	(0.57) (0.67) (0.60)		
Saldana et al. (2017)	13 (10F, 3M)	8−81.4 (6.25) 5−78.4 (9.37)	Game	Low	Stationary	<10	8	Visit 1								
Schmitz et al. (2018)	35 (19F, 16M) 10 (2F, 8M)	24.97 (3.71) 23.40 (3.2)	Game	Low	Walking		23 0	Exp 2 Post 1 Exp 3 Post 1							48.92 8.31	(45.92) (12.51)
Serge and Fragomeni (2017)	24 (12F, 12M)	19.75 (2.21)	Game	High	Walking	≥10	n/a n/a n/a n/a n/a n/a n/a n/a n/a	S1—Head Tilt S1—Head-Turn S1—Controller S2—Head Tilt S2—Head-Turn S2—Controller S3—Head Tilt S3—Head-Turn S3—Controller							6.55 8.23 5.61 13.56 13.84 15.58 21.04 20.57 10.60	(11.61) (9.13) (8.77) (18.32) (13.66) (13.26) (25.21) (18.51) (3.68)
Sharples et al. (2008)	19 (9F, 10M)	n/a	Game	Low	Controller	≥20	n/a	Experiment 1: HMD	28.62	(24.63)	27.53	(22.35)	32.97	(31.54)		
Singla et al. (2017)	30 (15F, 15M) 30 (15F, 15M)	25.62 25.62	360	Low	Stationary	≥10	0 0	HTC Vive Oculus Rift								
Song (2017)	14		360	High	Stationary	<10	n/a n/a	Control Experiment	63.70 35.40	(16.00) (12.20)	48.20 41.20	(11.20) (7.90)	83.50 37.80	(16.40) (14.30)		
Stanney et al. (2003)	240 240 240 240 (396F, 564M)	15−53 (range)	n/a	n/a	Controller	≥10 ≥20 ≥20 ≥20	13	15 min 30 min 45 min 60 min	15.86 21.50 23.49 24.80	(23.94) (24.85) (26.50) (25.04)	14.75 19.30 22.55 27.13	(20.19) (19.32) (20.18) (21.86)	24.07 28.94 32.54 33.47	(34.04) (35.67) (35.49) (34.43)	19.96 25.73 29.08 32.10	(25.92) (25.73) (26.63) (26.56)
St. Pierre et al. (2015)	120 (64F, 56M)	n/a	360	Low	Stationary	≥10	0 7 7 23	Baseline condition Constant condition Fixed amplitude condition Varying amplitude condition							24.19 27.43 34.53 60.84	(24.55) (32.87) (33.52) (41.22)
Stauffert et al. (2018)	45 (36F, 9M)	21.18 (2.58)	Min.	Low	Walking	<10	n/a	No latency	13.83	(17.91)	17.43	(14.15)	13.22	(15.95)	17.58	(15.78)
		21.18 (2.58)					n/a	With latency jitter	14.99	(15.84)	16.96	(16.93)	20.55	(17.94)	19.77	(16.69)
Tyrrell et al. (2018)	20 (9F, 11M)	46.5 (12)	Game	Low	Controller	<10	n/a	Control	24.33	(30.90)	12.13	(17.80)	23.66	(37.00)	21.88	(29.70)
Walch et al. (2017)	20 (5F, 14M)	25 (3.2)	Game	High	Controller	≥20	0								29.09	(27.65)
Weidner et al. (2017)	94 (24F, 70M)	24.8 (4.7)	Game	High	Controller	≥10	n/a								30.91	(28.24)
Young et al. (2014)	13 (7F, 6M)	18−23 (range)	Scenic	Low	Walking	<10	15									

*F, Female; M, Male; HMD, Head-mounted display; DoF, Depth of field; VR, Virtual reality*.

### Dropouts

The mean dropout rate reported across 46 experiments due to VR sickness was 15.6%. If studies did not report dropouts, they were not included in this analysis as it was unknown whether there were no instances of dropouts or whether they were just not reported.

### Description of Studies

Out of the 55 papers included in this review, 20 papers reported both subscale scores and total SSQ scores, 7 papers reported subscale SSQ scores only, and 16 papers reported total SSQ scores only. Twenty papers that reported SSQ scores also reported dropout rates. A further 12 papers that used the SSQ but only reported dropout rates were also included. The total number of experiments from these papers included 54 that reported the total SSQ scores and 38 that reported the subscale SSQ scores (these numbers include between group studies from the same paper). The number of participants included in all experiments represented 3,016 participants. Heterogeneity was consistently high for all analyses (*I*^2^ > 90).

Studies came from: Australia (*n* = 3), Canada (*n* = 1), Columbia (*n* = 1), Cyprus (*n* = 1), Finland (*n* = 1), Germany (*n* = 11), Greece (*n* = 1), Japan (*n* = 1), Korea (*n* = 4), Netherlands (*n* = 3), New Zealand (*n* = 1), Portugal (*n* = 1), Slovenia (*n* = 1), Spain (*n* = 1), United Kingdom (*n* = 2), United States of America (*n* = 22).

The pooled mean age of participants was 24 years (of 45 studies that included mean age), with the youngest sample having a mean age of 19.5 years and the oldest having a mean age of 80 years. Fifty-one studies included both female and male participants, 4 studies did not report sex distributions, and 41% of participants were female. Bivariate correlations between the SSQ and percentage of females in studies were not significant (*r* = −0.172, *p* = 0.170).

See Table 2 for a summary of results showing factors associated with both total and subscale SSQ scores.

**Table 2:** Summary of results.

	**Total SSQ score**	**Nausea**	**Oculomotor**	**Disorientation**
Content	*Q*-value = 18.745 *p* < 0.001	*Q*-value = 47.425 *p* < 0.001	*Q*-value = 59.106 *p* < 0.001	*Q*-value = 41.835 *p* < 0.001
Visual stimulation	*Q*-value = 0.491 *p* = 0.483	*Q*-value = 0.768 *p* = 0.381	*Q*-value = 7.314 *p* = 0.007	*Q*-value = 3.484 *p* = 0.062
Locomotion	*Q*-value = 15.987 *p* < 0.001	*Q*-value = 13.141 *p* = 0.001	*Q*-value = 18.893 *p* < 0.001	*Q*-value = 5.918 *p* = 0.052
Time	*Q*-value = 5.433 *p* = 0.066	*Q*-value = 30.362 *p* < 0.001	*Q*-value = 2.912 *p* = 0.233	*Q*-value = 7.126 *p* = 0.028
VR sickness condition	*Q*-value = 12.236 *p* < 0.001	*Q*-value = 29.059 *p* < 0.001	*Q*-value = 24.206 *p* < 0.001	*Q*-value = 6.562 *p* = 0.010
Age	*Q*-value = 7.430 *p* = 0.006	*Q*-value = 0.290 *p* = 0.590	*Q*-value = 0.010 *p* = 0.919	*Q*-value = 4.426 *p* = 0.035

*SSQ, Simulator Sickness Questionnaire; VR, Virtual reality*.

## Discussion

The aim of the review was to synthesize the literature on VR sickness symptoms using HMDs measured using the SSQ. The primary aim was to examine if VR sickness symptoms are influenced by content (four categories), the amount of visual stimulation, how a person moves in the virtual environment and exposure times. With a secondary aim of examining the influence of user characteristics (i.e., age and sex).

### SSQ Scores Interpretation

In this review, total SSQ mean scores ranged from 14.30 to 35.27. Pooled total SSQ scores were relatively high across all studies and content type (*M* = 28.00) with high levels of heterogeneity. Historically the SSQ was intended for military personnel using simulators, however, the different applications and interpretation of the scores have changed with increased use of VR and advancements in technology. When interpreting total SSQ scores, according to Kennedy et al. (2003); scores between 10 and 15 indicate significant symptoms; between 15 and 20 are a concern; and scores over 20 indicate a problem simulator. These cut-off scores were established from military personnel using flight simulators, these scores may differ in the general population, additionally, SSQ scores do tend to be higher in other virtual environments compared to flight simulators (Stanney and Kennedy, 1997; Kennedy et al., 2003). According to the Kennedy et al. (2003) categories, even the lowest total SSQ mean score of 14.30 found in studies including older adults in this current review would be regarded as significant symptoms. All remaining classifications displayed higher means with the highest total SSQ score displayed in studies that set out to induce motion sickness.

### VR Sickness Symptom Profiles

Across all studies, this review found the highest pooled SSQ subscale scores for disorientation (*M* = 23.50), followed by oculomotor (*M* = 17.09) and nausea (*M* = 16.72). This subscale distribution demonstrates the difference with the symptom profile of motion sickness where nausea typically has the highest rating, followed by oculomotor and disorientation (Rebenitsch and Owen, 2016). These findings increase awareness of symptoms that may be more likely to develop when using HMDs (e.g., dizziness, blurred vision and difficulty focusing). However, the weighting of these subscales makes it unclear as to what degree these symptoms differ.

### VR Content

The content characteristics in Figure 2 highlight the distinguishing features of the four content types that may account for the distribution of SSQ scores in this review. SSQ scores were significantly influenced by content type with gaming content displaying the highest total SSQ mean (*M* = 34.26). This effect was also seen for subscale SSQ scores with all measured subscale symptoms of nausea, oculomotor and disorientation highest for gaming content compared to other types of content (see Table 4). Consistent with these results, previous studies using gaming content reported the highest dropout rates, ranging from 44 to 100% (Merhi et al., 2007; Dennison et al., 2016; Munafo et al., 2017). The second highest total SSQ means were found in studies using 360 videos. This was followed by minimalist content, with scenic content producing the lowest total SSQ mean. The total SSQ means did not always correspond with dropout rates, for example higher dropout rates were found in scenic content than 360 videos. This discrepancy highlights the variability in how users tolerate HMD use that could be due to other factors. Exposure time, user characteristics or the amount of visual stimulation are all other factors that may have contributed to the high heterogeneity found in this review. Thus, a limitation of this current meta-analysis and meta-analyses in general is that methodological differences between studies are collapsed when pooling results.

### Influence of Visual Stimulation on Sickness

Content varies not only by type but also by the amount of visual stimulation offered. For example, all four types of content examined in this review may provide varying degrees of visual movement to the user. Oculomotor subscale SSQ mean scores were significantly higher for high visual stimulation compared with low visual stimulation. Some of the symptoms in the oculomotor subscale relate to eyestrain, difficulty focusing, difficulty concentrating and blurred vision. Despite recent improvements in display technology, stereoscopic HMDs may produce more side effects due to the vergence-accommodation conflict. Vergence refers to the way the eyes move laterally to adjust to items moving toward and away from the eyes combined with the process of focusing (accommodation). These visual processes do not occur naturally in a stereoscopic display as accommodation occurs at a fixed screen depth (Terzić and Hansard, 2016). This conflict may be a reason for the higher SSQ means for high visual stimulation in the oculomotor SSQ subscale. When there is a high level of visual stimulation there are more changes in the stimulus distance compared to content with low visual stimulation. The level of visual stimulation is meaningful, as research examining rapid vs. slow changes in stimulus distance found rapid changes to increase visual discomfort (Kim et al., 2014). In a virtual environment, a conflict may be created due to the differences in what a person sees and what their body experiences. With the emergence of new VR technologies, high-quality stereoscopic HMDs are now capable of simulating the visual and spatial properties of the real-world. Despite improvements, current technology still falls short of replicating how humans see and perceive depth under natural viewing conditions (Howarth and Costello, 1997). There are software solutions that can help to reduce discomfort by introducing blurring during motion (Budhiraja et al., 2017), however, this technique may not be effective for everyone. The shortcomings of current HMDs can produce unnatural visual conflicts, which have been shown to play a role in VR sickness (Carnegie and Rhee, 2015), especially when they are combined with visually stimulating VR environments (Kim et al., 2014).

### Locomotion Type in Virtual Environment

SSQ scores were significantly influenced by locomotion type with controller-based movement displaying the highest total SSQ mean (*M* = 32.55). Both nausea and oculomotor subscale SSQ scores means were also significantly influenced by locomotion type with higher scores when stationary as opposed to both controller-based moving and walking (see Table 4), high heterogeneity between studies has contributed to these differences. There are several other factors that can account for differences between total and subscale SSQ scores for locomotion between controller-based and stationary content. This includes differences in the number of studies, with seven stationary and five walking studies that reported subscale SSQ data, compared with 12 studies that reported total SSQ data for these locomotion categories. Additionally, relatively high total SSQ scores were reported for controller-based studies (Merhi et al., 2007; Budhiraja et al., 2017; Ragan et al., 2017) that did not report any subscale scores. Finally, these differences between SSQ totals and subscales may result from certain methods of locomotion having a greater impact on specific symptoms in the subscale SSQ scores depending on locomotion type that would not be reflected in the total SSQ scores. For example, being stationary in the real world may induce a greater conflict in a virtual environment where there is movement and hence may increase nausea symptoms. This is consistent with research that indicates a reduction in symptoms when user-initiated movement is matched to the environment (Lee et al., 2017; Misha et al., 2018), these findings also support the sensory conflict theory relating to a visual-vestibular conflict (Reason and Brand, 1975). Thus, the visual-vestibular conflict may be exacerbated by the type of content (moving vs. static) being viewed combined with the locomotion method. A reduction in visual-vestibular conflict may be the reason that the lowest total and subscale SSQ scores for locomotion were consistently reported in studies that included physically walking content. More research is needed to increase the understanding of how the type of locomotion can influence specific symptoms of VR sickness.

### VR Exposure Time on VR Sickness

**Figure 3:**
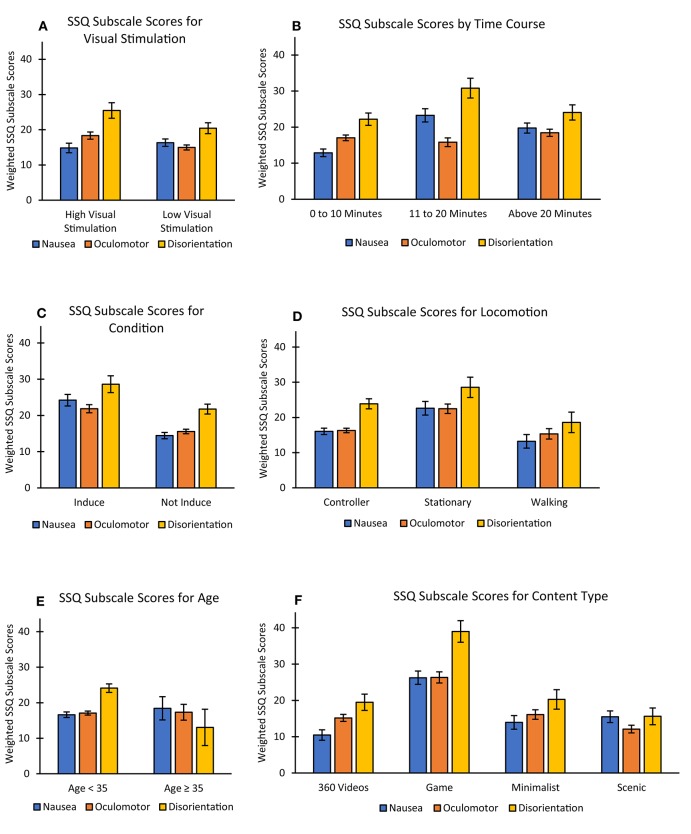
VR sickness symptoms across the different SSQ subscales of all measured factors including **(A)** Visual Stimulation, **(B)** Time, **(C)** VR Sickness Condition, **(D)** Locomotion, **(E)** Age, and **(F)** Content. SSQ, Simulator Sickness Questionnaire; Error bars represent standard error.

Both nausea and disorientation SSQ subscale scores in studies for exposure times of <10 min were lower than those that were equal to or >10 min. Interestingly scores were lower for studies that were equal to or >20 min than those equal to or >10 min (see Figure 3). This contradicts a recent summary in a review suggesting that longer exposure times are more likely to increase VR sickness (Duzmanska et al., 2018). Content may have been a factor contributing to this pattern of results within each of the time categories. In examining the distribution of content among the time breakdowns ≥10 min studies did have the highest percentage of gaming content (62%), compared to studies with the shortest (<10) and longest exposure times (≥20). In addition to this 50% of studies with the longest exposure times (≥20) consisted of minimalist or scenic content. More research is needed to determine the relationship between content and exposure time. Within-subject designs with different exposure times and controlled content may assist with answering questions around safe exposure times as this information is important when planning clinical trials to avoid VR sickness and dropouts and establish safe use procedures.

### Age and VR Sickness

Four studies included older samples (studies with a mean age range ≥35 years; *n* = 64) that reported total SSQ scores. Not only did these studies report lower total SSQ scores for older samples (*M* = 14.30) compared to younger samples (*M* = 28.44), these studies reported the lowest SSQ scores when compared with all other examined factors (see Table 3). Two of the four studies with older samples also included subscale SSQ scores with 37 participants in total. The disorientation subscale recorded significantly lower SSQ scores for the older samples compared with the younger samples. While scores for nausea and oculomotor subscales were higher for the older adult samples compared with younger samples, they were not statistically significant. Previous research has found inconsistent findings when looking at older samples (Kennedy et al., 2010; Benoit et al., 2015). Even though age has been reported as a user characteristic likely to predict motion sickness (Golding, 2006), the results from this review support previous research that there may be a decline in susceptibility to VR sickness as a person ages (Paillard et al., 2013). However, as there are a limited number of studies including older samples, these results should be interpreted with caution. Additionally, three of the studies used scenic content and one study used gaming content. What also needs to be considered is that the VR content for the studies including older adults may be assessing specific symptoms, and the virtual environments may be designed to reduce the likelihood of side effects. For example, two of the studies (Parijat and Lockhart, 2011; Kim et al., 2017) involved walking on a treadmill to assess gait or balance and consisted of content with the lowest total SSQ mean scores in this review (scenic content). It is also possible that older adults may experience symptoms that differ to younger adults as indicated with lower disorientation subscale SSQ scores found in the older samples (symptoms related to dizziness, vertigo, blurred vision, nausea and difficulty focusing). With many companies offering VR services to aged care facilities (Aged Care Virtual Reality, 2018; Reminiscience, 2018; Rendever, 2018), the use by older adults will continue to increase. Moreover, VR delivered in HMDs is being widely used for rehabilitation, assessment and even prediction of cognitive impairments in older adults (Optale et al., 2010; Corriveau Lecavalier et al., 2018; Howett et al., 2019). Therefore, more research is needed to evaluate safety aspects of using HMD-delivered VR with older adults having cognitive decline or other age-related health conditions.

**Table 3:** Simulator sickness questionnaire total scores.

		**Total SSQ**	
	***n***	***M (SE)***	**95% CI**
**Content**			
360 Videos	10	27.418 (3.434)	[20.688, 34.148]
Game	25	34.259 (2.392)	[29.571, 38.948]
Minimalist	5	21.709 (4.768)	[12.364, 31.055]
Scenic	10	17.329 (3.338)	[10.787, 23.871]
**VS**			
High	17	30.837 (3.597)	[23.787, 37.888]
Low	33	27.763 (2.511)	[22.841, 32.685]
**Locomotion**			
Controller	31	32.545 (2.174)	[28.284, 36.806]
Stationary	12	28.036 (3.417)	[21.338, 34.733]
Walking	12	16.993 (3.227)	[10.669, 23.317]
**Time**			
<10min	15	23.466 (3.226)	[17.144, 29.788]
≥10min	20	33.417 (2.905)	[27.723, 39.111]
≥20min	16	27.354 (3.121)	[21.238, 33.470]
**VR sickness condition**			
Induce	19	35.274 (2.700)	[29.983, 40.565]
Not induce	35	23.763 (1.882)	[20.075, 27.451]
**Age**			
Mean <35	50	28.438 (1.521)	[25.457, 31.420]
Mean ≥ 35	4	14.299 (4.959)	[4.579, 24.019]
**All studies**	54	28.001 (1.706)	[24.656, 31.345]

*VS, Visual stimulation; VR, Virtual reality*.

**Table 4:** Simulator sickness questionnaire subscale scores.

		**Nausea**		**Oculomotor**		**Disorientation**	
	***n***	***M (SE)***	**95% CI**	***M (SE)***	**95% CI**	***M (SE)***	**95% CI**
**Content**							
360 Videos	7	10.460 (1.437)	[7.644, 13.276]	15.186 (0.974)	[13.278, 17.095]	19.493 (2.246)	[15.091, 23.895]
Game	13	26.261 (1.832)	[22.671, 29.852]	26.336 (1.540)	[23.318, 29.353]	38.999 (2.980)	[33.158, 44.840]
Minimalist	6	13.944 (1.874)	[10.271, 17.617]	16.118 (1.306)	[13.559, 18.677]	20.270 (2.686)	[15.005, 25.534]
Scenic	8	15.513 (1.597)	[12.383, 18.642]	12.106 (1.066)	[10.017, 14.196]	15.610 (2.312)	[11.078, 20.142]
**VS**							
High	12	14.830 (1.359)	[12.167, 17.493]	18.339 (1.017)	[16.346, 20.333]	25.473 (2.212)	[21.138, 29.808]
Low	22	16.338 (1.055)	[14.271, 18.404]	14.968 (0.721)	[13.555, 16.380]	20.435 (1.547)	[17.402, 23.467]
**Locomotion**							
Controller	27	16.040 (0.907)	[14.262, 17.817]	16.303 (0.643)	[15.043, 17.562]	23.875 (1.424)	[21.084, 26.666]
Stationary	7	22.609 (1.936)	[18.815, 26.403]	22.470 (1.345)	[19.835, 25.106]	28.552 (2.883)	[22.903, 34.202]
Walking	5	13.208 (1.920)	[9.446, 16.970]	15.336 (1.481)	[12.433, 18.238]	18.599 (2.909)	[12.898, 24.300]
**Time**							
<10min	18	12.884 (1.066)	[10.795, 14.973]	17.030 (0.791)	[15.480, 18.579]	22.217 (1.713)	[18.858, 25.575]
≥10min	10	23.281 (1.836)	[19.683, 26.880]	15.819 (1.206)	[13.455, 18.183]	30.827 (2.745)	[25.446, 36.208]
≥20min	9	19.748 (1.404)	[16.995, 22.500]	18.442 (0.996)	[16.490, 20.393]	24.078 (2.103)	[19.955, 28.200]
**VR sickness condition**							
Induce	13	24.211 (1.590)	[21.093, 27.328]	21.859 (1.116)	[19.671, 24.047]	28.662 (2.326)	[24.102, 33.222]
Not Induce	25	14.436 (0.871)	[12.730, 16.143]	15.574 (0.621)	[14.357, 16.791]	21.755 (1.363)	[19.084, 24.427]
**Age**							
Mean <35	36	16.613 (0.789)	[15.067, 18.158]	17.078 (0.565)	[15.971, 18.185]	24.095 (1.207)	[21.729, 26.461]
Mean ≥ 35	2	18.423 (3.269)	[12.015, 24.831]	17.313 (2.234)	[12.935, 21.691]	13.049 (5.110)	[3.034, 23.065]
**All studies**	38	16.723 (0.768)	[15.218, 18.229]	17.092 (0.547)	[16.019, 18.165]	23.499 (1.173)	[21.200, 25.797]

*VS, Visual stimulation; VR, Virtual reality*.

### Sex and VR Sickness

An analysis of sex differences was performed with a correlation between the percentage of females in studies and total SSQ scores. Sex breakdown was not supplied in studies when reporting total SSQ scores, therefore, this was the only way that sex could be analyzed and therefore a limitation of this analysis. The results indicated no difference. This is not consistent with research indicating that females are at higher risk of VR sickness (Lawson et al., 2004). Finding evidence in studies that females are more susceptible than males to VR sickness depends on what study is examined with many confounding variables not taken into account (Lawson, 2015). The importance of this topic suggests that more research is needed to better understand the incidence of VR sickness based on sex differences. Age and sex have been stated as being the most common user characteristics likely to predict motion sickness (Golding, 2006) highlighting a need for further research. Other user characteristics including ethnicity; motion sickness susceptibility; fitness; and prior experience of VR may provide a deeper insight into symptomatology of user characteristics and assist to develop a more targeted approach to dealing with VR sickness.

### Strengths and Limitations

This is the first study to pool estimates of VR sickness symptoms measured with the SSQ using HMDs with a pooled sample size of 3,016, however, the study is not without limitations. Although the most commonly used measure of VR sickness was used (SSQ), there were also many studies excluded (112) that did not use the SSQ. As the SSQ is self-report participants may under or over-report symptoms. Physiological measures can assist with overcoming this limitation however, a consensus is yet to be reached on the best physiological response for assessing VR sickness (Duzmanska et al., 2018). The scoring system for the SSQ can create some confusion and this was seen in this review with some authors incorrectly calculating total scores. Another limitation of the SSQ is the relevance of symptoms for HMD use. For example, the *Virtual Reality Symptom Questionnaire* (Ames et al., 2005), increased the focus on oculomotor symptoms, while Kim et al. (2018) removed the symptom of nausea in the *Virtual Reality Sickness Questionnaire*, due to not contributing to motion sickness compared with other symptoms, both of these studies were HMD specific. For a more detailed discussion of alternative measures see (Hale and Stanney, 2014).

Additionally, all analyses had high heterogeneity demonstrating large variation across the included studies. As well as individual differences of age and sex, susceptibility to VR sickness can also vary between individuals and therefore influence results. Gaming or VR experience is another individual difference that can influence the likelihood of side effects and needs to be both reported and taken into account during analysis of results. The small number of studies including older adults and lack of reporting of sex differences and dropouts are also limitations and areas requiring further research or improved reporting in future VR studies including HMDs. As 22 studies did not report dropout rates, the rate of 15.6% may be inflated if many of these studies did not have dropouts, however, we cannot assume there were no dropouts if they were not reported. This highlights the need to make reporting of dropout rates a standard in VR research.

Finally, another limitation involves the varied nature of the HMDs used across these studies. HMDs can differ in terms of field of view, use of stereo, resolution, framerate, availability of inter-pupillary distance controls/adjustment, and other technical display factors. Modern HMDs from the last 5 years differ fundamentally from the more limited display technology that was available before these recent advances (Kourtesis et al., 2019), and since 35% of papers included in this analysis used these older HMDs, it is difficult to predict how those findings would predict the occurrence of symptoms with use of currently available HMDs. Moving forward, there is an obvious need for more controlled laboratory research with standard reference VR environments that are adjustable in terms of content, movement, user interaction, etc. With such specifically created environments, one would be able to test out the incidence of side effects across different display types with varied hardware capabilities. This will be essential for promoting parametric research that creates a database of known properties for different types of virtual environments delivered across varied hardware types and would serve to produce the baseline normative data needed to enable better research in how to mitigate or eliminate the incidence of these use-limiting side effects.

### Conclusion

Previous research has focused on the influence of technological aspects on VR sickness. This review advances this knowledge by examining content as a major contributing factor to VR sickness, which will remain a problem despite future technological advances. Our findings show that content significantly influences VR sickness symptoms. Recent HMD technology can provide a better experience (Kourtesis et al., 2019) and if this is combined with careful selection of content the risk of VR sickness can be reduced and those symptoms that do occur can be easily managed. In this review, we compared our total SSQ scores with the cut-off scores suggested by Kennedy et al. (2003), what these scores mean in relation to HMDs and how these scores relate to the general population remains unclear. Nevertheless, comparing total scores between studies shows that content is a major contributing factor. This review also highlights the need for a further understanding of the influence of user characteristics such as age and sex as there is a lack of studies including older samples, and sex differences that are often not reported. Increasing our understanding of VR sickness could be particularly valuable to researchers and practitioners, as there may be ethical and liability implications in research, training and clinical applications.

## Data Availability Statement

The datasets generated for this study are available on request to the corresponding author.

## Author Contributions

DS, AS, and TL conception of the work and analyzed and interpreted the results. DS and AS article selection and screening. DS wrote the manuscript. All authors revised the work critically for important intellectual content and have read and approved the manuscript. AS and DS created Figure 2, certain 3D models for this figure sourced from cadnav.com and modified.

### Conflict of Interest

The authors declare that the research was conducted in the absence of any commercial or financial relationships that could be construed as a potential conflict of interest.
